# Eye Movement Desensitization and Reprocessing (EMDR) re-examined as cognitive and emotional neuroentrainment

**DOI:** 10.3389/fnhum.2014.01035

**Published:** 2015-01-07

**Authors:** Olivier A. Coubard

**Affiliations:** The Neuropsychological Laboratory, CNS-FedParis, France

**Keywords:** anxiety disorders, Post-Traumatic Stress Disorder, Eye Movement Desensitization Reprocessing, neuroentrainment, attention, emotion

Jessica Phillips-Silver and colleagues recently introduced a new concept, neuroentrainment, to refer to the human tendency for synchronization in time and affect through coordinated rhythmic movements. Entrainment, from French *entraîner*, originally refers to the spatiotemporal coordination between several individuals in response to a rhythmic signal (Phillips-Silver and Keller, 2012). By extension, the neuroentrainment framework by Jessica Phillips-Silver and colleagues aims at developing theoretical and technical tools for further understanding how entrainments from different movement disciplines favors body and mind development of healthy volunteers and may treat patients suffering from various pathological conditions. In cognitive and behavioral (CBT)-inspired therapies, clinicians have a theoretical and technical tool so-called Eye Movement Desensitization and Reprocessing (EMDR) to treat anxiety disorders, particularly Post-Traumatic Stress Disorder (PTSD). Knowing that EMDR can treat affective disorders through coordinated movements, we examine in this opinion article the possibility that EMDR may act as neuroentrainment.

## EMDR as cognitive neuroentrainment

In 1987, Francine Shapiro observed on herself cognitive and emotional changes after she had made rhythmic left-right smooth pursuit movements of the eyes. Rather than being focused on negation cognition and worried, movement had switched her state-of-mind to new perspective and hope. From that observation, she developed a cognitive and behavioral (CBT)-inspired protocol called EMDR to treat PTSD (Shapiro, 1989a,b). EMDR may act as cognitive neuroentrainment. One goal of EMDR is to alleviate negative cognition during the desensitization phase and to replace it by positive cognition during the reprocessing phase. To achieve this goal, many aspects of patient's cognition are stimulated and entrained. First, the EMDR therapist performs functional analysis to identify a single traumatic memory related to a particular event. Traumatic memory is here defined as a blend of multi-sensory images (visual, auditory, olfactory, somatosensory, related to taste), negative cognition, negative emotion, and their related unpleasant physical sensations (Van Der Kolk, 1994; Van Der Kolk and Fisler, 1995). Once traumatic memory is identified, the patient is invited to (re)build the visual image associated with its most acute moment. Thus, visual mental imagery is demanded to provide the clearest visual image, which may vary in nature and intensity throughout the procedure. Then the patient is asked to identify the negative cognition he/she may express about him/herself while thinking about the scene, and to quantify his/her belief in such cognition on a subjective scale. Such exercise requires multiple cognitive abilities: attention, self-consciousness or feeling of self, personal semantic memory, and metacognition (debriefings during which the patient is asked to communicate on his/her thoughts and feelings), in order to identify potential dysfunctional schemes related to the self (Young, 1999; Young et al., 2002). Later in the reprocessing phase, the patient is requested to replace such negative cognition by a new positive one. This cognition is reinforced through rhythmic movements and acts as a new narrative content of self-consciousness (Meichenbaum and Fitzpatrick, 1993). From the desensitization phase to the reprocessing one, the patient goes through multiple cognitive steps, in which episodic memory (memory of well-defined events in space and time) is also highly required. EMDR is indeed concrete in the sense that it only applies to well-defined events in patient's experience of time and space. PTSD is made up with a node linking different events (where the identified traumatic memory is only one of them), which may be disseminated in space and time of patient's life, but which EMDR is able to gather as free association of psychoanalysis (Wachtel, 2002). Indeed during the EMDR procedure, the patient jumps from one episodic memory to another. However EMDR allows faster processing than psychoanalysis due to the media used—rhythmic movement in the former vs. language in the latter –presumably because the neural substrate of rhythmic movement has more direct links to the limbic system than that of language areas (see below). Once disseminated fragments of traumatic memory have been reconnected, they can be integrated into a new personal semantic memory network (Braun, 1988; Van Der Kolk et al., 2001). Integration of traumatic memory disseminated/dissociated fragments into new cognitive schemes is the main goal of EMDR (Van Der Kolk, 1994; Van Der Kolk and Fisler, 1995; Shapiro, 2001). Throughout the EMDR procedure, all aspects of attention are also demanded: arousal (or vigilance), selection, and control (Pashler, 1998; Parasuraman, 2000). Distraction, as a feature of attention, has been advocated to partially account for EMDR effects, allowing the patient to be in the intermediate situation in which he/she is not distracted enough and not capable of negative association (Dyck, 1993). Switching between periods of exposure, movements of the eyes, and metacognition also requires cognitive flexibility (Lohr et al., 1998; McNally, 1999). Taken together, EMDR may act as cognitive neuroentrainment as its procedure requires several cognitive demands: attention, in particular attentional control, but also episodic and personal semantic memory, visual mental imagery, self-consciousness, and metacognition. As compared to psychoanalysis, less importance is given to language, which is restricted to expedient and concise descriptions of thoughts and feelings.

## EMDR as emotional neuroentrainment

EMDR primarily acts as emotional neuroentrainment since it aims at treating PTSD, an anxiety disorder. During the desensitization phase of the protocol, left-right smooth pursuit eye movements have also the benefit to alleviate negative emotion and their related unpleasant physical sensations (Shapiro, 1989a,b, 2001). To achieve this goal, the patient is first invited to identify the negative emotion associated with traumatic memory, which may be fear or anger in most cases. Negative emotion is usually consistent with the negative cognition revealing dysfunctional scheme about the self. Once the emotion is identified, the patient is then requested to describe the physical sensation(s) associated with this negative emotion. Such sensation might be some pain in the stomach or some unpleasant sensation in the throat. In any case, identifying as precisely as possible the physical sensation associated with negative emotion and quantifying the level of disturbance on a subjective scale help the patient to make sense of his/her emotional state (Gendlin, 1996). It once again provides both the patient and the therapist concrete information to assess and modulate the level of anxiety throughout the procedure (Shapiro, 2001). This aspect of the protocol is crucial as it participates to develop the patient's “metaemotion”—the ability to think about one's own emotions and potentially others' ones. During the EMDR procedure, the switch between periods of exposure and metaemotion forces the patient to face his/her negative emotion thus preventing its avoidance (Shapiro, 2001). Such switch reinforces self-mastery and self-efficacy (Bandura, 2000). Finally, EMDR is also compatible in its procedural aspects with oriental meditative practice (Kabat-Zinn, 1990; Krystal et al., 2002), full attention/consciousness of behavioral therapies (Linehan, 1993), and acceptance/commitment therapies (Hayes et al., 1999). The common denominator of these theoretical and practical frameworks is to reach some balance in the attentive feeling of self, which may be defined as harmonious flow of sensory, cognitive, emotional, and physical associations (Servan-Schreiber, 2003). The best results in EMDR are obtained in the intermediate state in which attention and emotion work fluently and in harmony. In other words, one goal of EMDR is to make sensitive to emotion the most attentive part of the mind—attentional control. Our suggestion is that rhythmic movements of the eyes, or potentially of other body parts, may facilitate such flow of attention and emotion. In terms of Bayesian models of decision (e.g., Schall, 2004), rhythmic movement *per se* is sufficient to decrease the distance to thresholds of excitatory units for movement, leading to release of inhibition (i.e., distraction) in the attentional network, and making dysfunctional cognitive and emotional information labile enough for their manipulation and integration. In this framework, time may be the core of cognitive and emotional processes, and EMDR may be viewed as re-mastering time, which has been lost with PTSD. Taken together, EMDR acts as emotional neuroentrainment as it develops emotional control, which involves at least four essential functions: (i) the ability to identify one's own emotional state and others' one; (ii) the ability to understand the natural course of emotions; (iii) the ability to reason and argue about one's own emotions and others' ones; and (iv) the ability to deal with and control one's own emotions and others' ones (Servan-Schreiber, 2003). About ability (ii), it is worth noting that EMDR does not contradict Freud's seminal description of the natural course of emotions, initially focused on one's own emotions before reasoning about others' ones (i.e., empathy) (Freud, 1917). Once again, the difference between EMDR and psychoanalysis is the media used—movement vs. language, respectively—and their temporal consequences, which leads us to our final issue about time and affect.

## EMDR may induce synchronization in time and affect

Since the original observation of Shapiro (1989a,b), over 300 studies have examined the effects of EMDR and several meta-analyses have shown its higher efficiency in PTSD as compared to pharmacological or other psychological interventions (e.g., Van Etten and Taylor, 1998). However, EMDR mechanism is hitherto unknown. We have mentioned that rhythmic movements of the eyes, originally obtained by left-right smooth pursuit in response to bilateral movement of a visual stimulus, are an important feature of EMDR protocol (Shapiro, 1994). Importantly, bilateral stimulation can be not only visual but also auditory (i.e., a sound stimulating ears bilaterally) or tactile (i.e., a bilateral stimulation of any body parts) (Shapiro, 1994, 2001). Thus, rhythmic and bilateral aspects of the sensory stimulation, regardless of the modality, seem to be more crucial than eye movements *per se*. How can EMDR be able to concomitantly induce synchronization in time and affect? Among hypotheses about EMDR effects, some authors have suggested that it may favor hemispheric synchronization. According to Shapiro (2001), normal neuropsychological condition is given by excitatory and inhibitory mechanisms in which positive and negative events are integrated by an adaptive information processing system (AIPS). Imbalance may occur whenever the AIPS is unable to integrate negative events, due to either insufficient capacity of AIPS or exceptional intensity of the event, resulting in traumatic memory. By stimulating the AIPS similarly to Rapid Eye Movement (REM) paradoxical sleep stage, EMDR in the awake patient might activate both hemispheres as a pacemaker thus facilitating down regulation of the limbic system and integration of dysfunctional information (Bergmann, 2000; Shapiro, 2001; Stickgold, 2002). Such assumption is supported by studies on hemispheric asymmetry in emotional processing (Heller et al., 1995; Keller et al., 2000). Functional brain imaging of emotional processing in PTSD and other anxiety disorders has evidenced hypoactivation in the dorsal and rostral anterior cingulate cortex (ACC) and ventromedial prefrontal cortex-structures linked to the regulation of emotion (Etkin and Wager, 2007). Additionally, a SPECT study showed that EMDR intervention in PTSD patients led to hyperactivation of ACC and left frontal lobe (Levin et al., 1999). How can synchronization in space (i.e., between two hemispheres) be related to some synchronization in time? Among the different visual systems, one of them is directly related to the circadian clock. Indeed some contingent of intrinsically photosensitive retinal ganglion cells (ipRGCs) is directed to the supra-chiasmatic nucleus in the hypothalamus (Dhande and Huberman, 2014). Through melanopsin, this retino-hypothalamic visual system projects to the pineal gland, which itself produces melatonin, and regulates various behavioral and biological functions and circadian rhythms related to temperature, wake/sleep, reproduction, autonomic, and hormonal functions (Trachtman, 2010). It is worth noting that ipRGCs have also been shown to influence mood, presumably via their inputs to the amygdala or habenula (Hattar et al., 2006; LeGates et al., 2012). Additionally M1 subtype of ipRGCs also targets the superior colliculus (SC), which is itself linked to the amygdala and the orbitofrontal cortex (Krolak-Salmon et al., 2004). Taken together, our suggestion is that visual bilateral stimulation may be able to induce (i) synchronization in time by stimulating the circadian clock through the retino-hypothalamic visual system; and (ii) synchronization in affect by stimulating the limbic system (i.e., amygdala and orbitofrontal cortex) directly or indirectly (via SC) targeted by ipRGCs. The way non-visual bilateral stimulation induces similar benefits may be obtained via supramodal cerebral areas such as the tectal platform. To conclude, EMDR is a rich theoretical and technical tool, whose neural mechanism needs to be further investigated. EMDR may act as cognitive and emotional neuroentrainment by inducing synchronization in time and affect, possibly through the retino-hypothalamic visual pathway and related supramodal subcortical and cortical systems, which are linked to the limbic system.

### Conflict of interest statement

The authors declare that the research was conducted in the absence of any commercial or financial relationships that could be construed as a potential conflict of interest.
